# Molecular Epidemiology of Hospital Outbreak of Middle East Respiratory Syndrome, Riyadh, Saudi Arabia, 2014

**DOI:** 10.3201/eid2111.150944

**Published:** 2015-11

**Authors:** Shamsudeen F. Fagbo, Leila Skakni, Daniel K.W. Chu, Musa A. Garbati, Mercy Joseph, Malik Peiris, Ahmed M. Hakawi

**Affiliations:** King Fahad Medical City, Riyadh, Saudi Arabia (S.F. Fagbo, L. Skakni, M.A. Gabrati, M. Joseph, A.M. Hakawi);; The University of Hong Kong, Hong Kong, China (D.K.W. Chu, M. Peiris)

**Keywords:** Middle East respiratory syndrome, MERS, Middle East respiratory syndrome coronavirus, MERS-CoV, coronavirus, viruses, transmission, molecular epidemiology, nosocomial infection, health care workers, phylogeny, mutation, respiratory infections, zoonoses, Saudi Arabia

## Abstract

We investigated an outbreak of Middle East respiratory syndrome (MERS) at King Fahad Medical City (KFMC), Riyadh, Saudi Arabia, during March 29–May 21, 2014. This outbreak involved 45 patients: 8 infected outside KFMC, 13 long-term patients at KFMC, 23 health care workers, and 1 who had an indeterminate source of infection. Sequences of full-length MERS coronavirus (MERS-CoV) from 10 patients and a partial sequence of MERS-CoV from another patient, when compared with other MERS-CoV sequences, demonstrated that this outbreak was part of a larger outbreak that affected multiple health care facilities in Riyadh and possibly arose from a single zoonotic transmission event that occurred in December 2013 (95% highest posterior density interval November 8, 2013–February 10, 2014). This finding suggested continued health care–associated transmission for 5 months. Molecular epidemiology documented multiple external introductions in a seemingly contiguous outbreak and helped support or refute transmission pathways suspected through epidemiologic investigation.

## Abstract 2

A contiguous outbreak was the result of multiple introductions from outside the hospital.

Middle East respiratory syndrome (MERS) coronavirus (MERS-CoV) was first recognized as a cause of severe human respiratory disease in 2012 (*1*). As of June 19, 2015, a total of 1,338 confirmed cases of MERS and at least 475 MERS-associated deaths had been reported (*2*). Human zoonotic infections have largely been acquired in the Middle East. Imported cases in Europe, North America, Africa, and Asia have been linked to travel to the Middle East, occasionally with local secondary transmission (*2*).

Although human infections are zoonotic in origin, clusters of human-to-human transmission have been reported, particularly within households or health care settings (*3**–**6*). In an outbreak in Jeddah, Saudi Arabia, in 2014 involving multiple health care facilities, 255 laboratory-confirmed MERS cases were documented during a 2-month period, but intensified infection prevention measures in hospitals terminated that outbreak (*6**,**7*). Available genetic data for these patients showed that they were clustered, which suggested widespread transmission of related viruses (*6*). Of 191 symptomatic patients, 40 were health care workers (HCWs). For the remaining patients for whom data were available, most had some form of contact with a health care facility or patients with suspected MERS. Investigation of outbreaks in health care settings also identified asymptomatic and milder cases, especially in healthy young adults and HCWs with no underlying illnesses (*7*). Dromedary camels have been proposed as a source of human infection; however, the possibility of other reservoirs and intermediate hosts has not been excluded (*2**,**8*).

Molecular epidemiologic analysis of transmission was attempted for a 2013 MERS outbreak at multiple health care facilities in the eastern region of Saudi Arabia (*5*). Combined analysis of genomic and epidemiologic data provided insights into transmission chains that would otherwise not have been apparent. The study on the 2014 Jeddah outbreak included analysis of viral sequences from 2 hospitals in Riyadh and identified a cluster of infections at the Prince Sultan Military Medical City (PSMMC) during March–April 2014 (*6*). In this study, we analyzed viral genetic data for patients and HCWs with MERS at King Fahad Medical City (KFMC), Riyadh, Saudi Arabia, during February 1–May 31, 2014, and available epidemiologic data to better understand transmission within the hospital and place the outbreak in KFMC in the context of contemporaneous MERS outbreaks in other hospitals in Riyadh.

## Materials and Methods

### Clinical Setting

KFMC is a 1,200-bed tertiary care hospital in Riyadh that comprises 4 hospitals and 4 medical centers on 1 campus. The main hospital is affiliated with specialized women’s, children’s, and rehabilitation hospitals. The 4 centers are the National Neuroscience, Heart, Oncology, and Diabetes centers. The main hospital, affiliated hospitals, and centers provide nationwide referral services. This study was approved by the Institutional Review Board of KFMC. 

The emergency department (ED) is located on the ground floor of KFMC. It accepts patients from throughout Saudi Arabia; in 2014, there were 139,173 recorded visits. Time in the ED is usually brief, but some patients might have extended ED stays depending on availability of isolation rooms in the wards.

**Figure 1:**
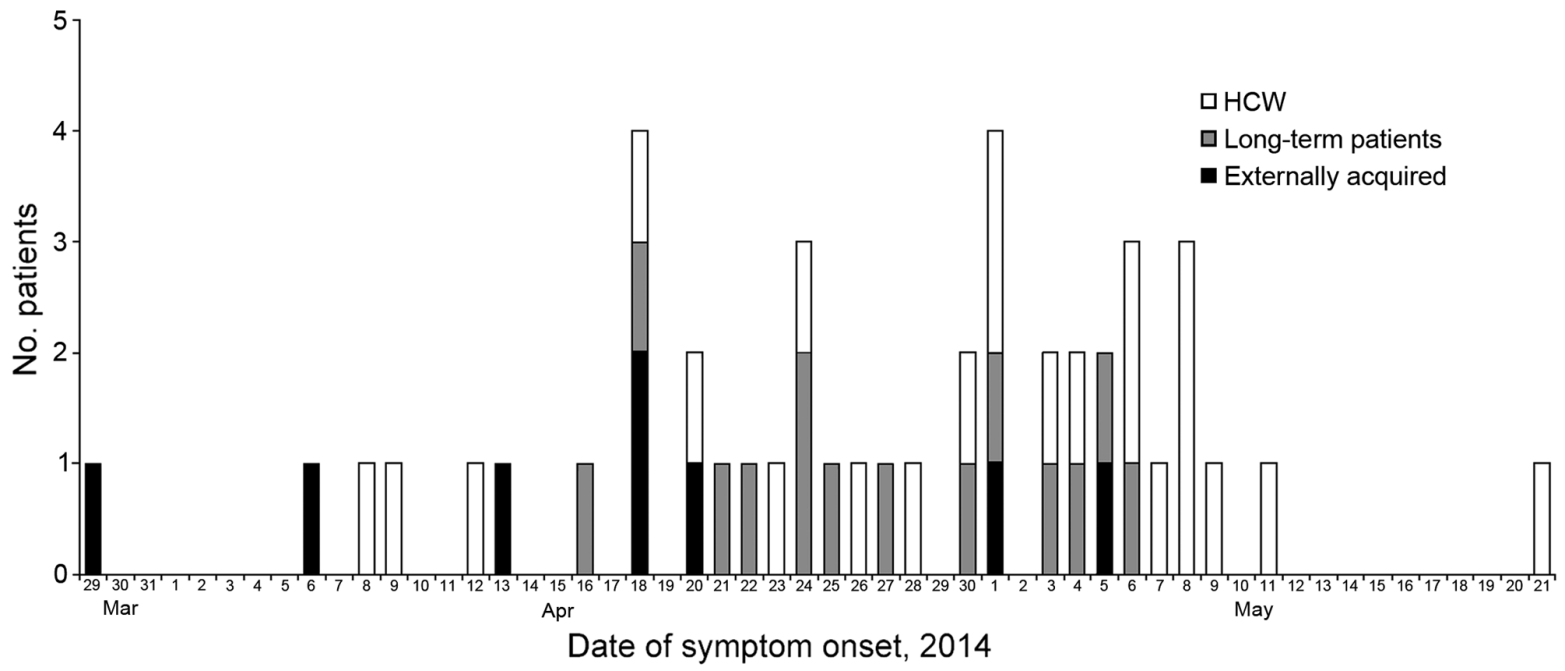
Date of symptom onset for patients with confirmed Middle East respiratory syndrome coronavirus (MERS-CoV) infection hospitalized at King Fahad Medical City, Riyadh, Saudi Arabia, 2014. For 4 asymptomatic health care workers (HCWs) detected by screening, date of virus detection, rather than symptom onset, is indicated.

Medical wards (MWs) MW-C and MW-D, which have 50 beds combined (Technical Appendix 1 Figure 1), are located in the main hospital and admit patients from the ED, outpatient clinics, and referrals from elsewhere in Saudi Arabia. Most rooms in these 2 adjacent wards have 4 beds. However, MW-C has 6 isolation rooms, 2 with negative pressure ventilation, and MW-D has 4 isolation rooms, none with negative pressure ventilation. Patients are occasionally moved between the 2 wards, but nurses work only in their assigned wards.

### Patients and Specimens

Patients, including HCWs, confirmed to have MERS diagnosed at KFMC during February 1–May 31, 2014, composed the study population. Nasopharyngeal swab specimens and tracheal aspirates or bronchoalveolar lavages were collected for viral diagnosis. A case of MERS, according to the Saudi Arabian Ministry of Health definition, was fever and acute respiratory illness in a patient who had a positive test result for MERS-CoV infection. Criteria for investigation of patients and HCW for MERS-CoV is provided in Technical Appendix 1.

### Laboratory Diagnosis

A reverse transcription PCR diagnostic kit (MERS-Coronavirus EMC Orf1a and SA1 EMC upstream E-gene, Light Mix Modular Assays; TIB MOLBIOL, Adelphia, NJ, USA, and Roche, Mannheim, Germany) was used for the screening and confirmation of MERS-CoV infection. Each sample was also tested simultaneously for 15 respiratory viruses (influenza A and B; parainfluenza viruses 1, 2, 3, and 4; respiratory syncytial virus; adenovirus; enterovirus; human metapneumovirus; human coronaviruses 229E, OC43, NL63 and HKU-1; and human bocavirus) by using the Seeplex RV15 ACE Detection Kit (Seegene Inc., Seoul, South Korea). Samples from the early phase of the outbreak were tested for MERS-CoV at the Ministry of Health laboratories; midway into the outbreak, KFMC developed in-house MERS-CoV testing capability.

### Epidemiologic Data

Patient demographics and epidemiologic data on study participants were collected by retrospective chart review, from electronic health records, and from leave or sick leave records of staff. Patients with confirmed cases of MERS were spatiotemporally mapped within the hospital. Additional contact histories were obtained through direct interviews with the infected HCWs or patients. On the basis of date of hospital attendance or admission, date of onset of illness, and reported incubation period for MERS (median 5 days, range 2–14 days) (*9*), the patients were classified into those acquiring infection outside KFMC (externally acquired), long-term patients acquiring infection while at KFMC (long-term patients) and HCWs working at the hospital. HCWs were presumed to have acquired nosocomial infections at KFMC, although infection outside the hospital could not be excluded.

Potential transmission links were identified on the basis of patients or HCW present or working in the same ward or ED concurrently with a MERS patient. Given the retrospective nature of this study, it was not possible to assess whether HCW exposures occurred without use of adequate personal protective equipment (PPE).

### Genetic Sequencing and Phylogenetic Analysis

cDNA was synthesized by using gene-specific primers for different regions of the MERS-CoV genome and subsequently subjected to multiple sets of PCR that covered the entire virus genome (primers available on request). Overlapping PCR products generated were sequenced by using MERS-CoV–specific primers. Sequences (without primer sequences) were aligned and assembled by using Geneious version 8.0.5 (http://www.geneious.com). Genomes were sequenced with >3–5 times coverage.

A time-resolved phylogenetic tree was estimated from a concatenated gene alignment of MERS-CoV genome by using BEAST version 1.8 (http://beast.bio.ed.ac.uk/). Analysis was conducted by using a general time-reversible model and gamma-distributed sites with separate rates for the 3 codon positions under a relaxed lognormal clock model.

## Results

### Descriptive Epidemiology

The number of specimen tested for MERS-CoV in March, April, and May 2014, were 3, 222 and 1,731, respectively, increasingly markedly during the course of the outbreak. During the study period, 45 patients at KFMC had virologically confirmed MERS. Eight of these patients had externally acquired infections, and 13 long-term hospitalized patients had nosocomial infections; 23 HCWs had MERS-CoV infections, presumably acquired at KFMC. Patient EA-9 (disease onset May 5, first ED visit May 1) might have been infected either at KFMC or at an external source.

Enhanced surveillance identified 4 asymptomatically infected HCWs. Disease onset dates of different patient groups are shown in Figure 1. Thirteen patients died of their infections: 3 of 8 patients with externally acquired infections, 9 of 14 long-term hospitalized patients, and 1 of 23 HCWs. MERS-CoV–infected HCWs had a median age of 35.5 years (range 24–58 years); non-HCWs had a median age of 60 years (range 12–77 years) (p<0.005). Demographic characteristics of all patients and work locations of infected HCWs are shown in Technical Appendix 1 Table 1.

### Viral Genetic Analysis

To investigate virus introduction and intrahospital transmission pathways, available archived respiratory specimens from 15 patients who had high viral loads were obtained and genetically analyzed. Whole-genome sequences were obtained from 10 patients and a partial genome was obtained from 1 patient (Table; Technical Appendix 1 Table 2) (GenBank accession nos. KT121572–KT121581 and KT202801).

**Table:** Characteristics of 21 patients tested for infection with MERS-CoV, King Fahad Medical City, Saudi Arabia, 2014*

Patient	Age, y/sex	Date of illness onset	Date of first ED visit	Date of hospitalization	Patient group	Outcome
Externally acquired infections						
EA-1	32/M	Mar 29	Apr 5	Apr 6	Patient	Recovered
EA-2	65/F	Apr 6	Apr 11	Apr 12	Patient	Deceased
EA-3	46/F	Apr 13	Apr 20	Apr 21	Patient	Recovered
EA-4	70/M	Apr 18	Apr 22	Apr 28	Patient	Deceased
EA-5	64/M	Apr 18	Apr 27	Apr 28	Patient	Recovered
EA-6	22/F	Apr 20	Apr 27	Apr 28	Patient	Recovered
EA-7	28/F	May 1	May 2	Transferred	Patient	Transferred
EA-8	21/F	May 5	May 8	May 9	Patient	Deceased
EA-9†	50/F	May 5	May 1	May 3	Patient	Deceased
Nosocomial infections						
KFMC-0	34/F	Apr 9	Apr 16	Apr 17	ED nurse	Recovered
KFMC-1	45/F	Apr 20	Apr 29	May 2	ED nurse	Deceased
KFMC-2	60/F	Apr 25	Apr 4	Apr 5	Patient	Deceased
KFMC-3	62/F	Apr 27	Feb 1	Jan 12	Patient	Deceased
KFMC-4	63/F	May 1	Apr 21	Apr 22	Patient	Deceased
KFMC-5	56/F	May 3	May 10	May 12	Nurse, MW-D	Recovered
KFMC-6	74/F	May 6	Mar 19	Mar 21	Patient	Transferred
KFMC-7	36/F	Apr 26	Apr 30	May 3	Nurse, MW-C	Recovered
KFMC-8	53/F	Apr 30	Mar 27	Mar 28	Patient	Recovered
KFMC-9	29/M	May 1	May 7	May 9	ED nurse	Recovered
KFMC-10	46/F	Apr 23	Apr 30	May 5	Nurse, MW-C	Recovered
KFMC-11	41/F	Apr 24	Apr 27	Apr 30	Nurse, MW-C	Recovered

*MERS-CoV, Middle East respiratory syndrome coronavirus; ED, emergency department; KFMC, King Fahad Medical City; MW, medical ward. †This patient visited the ED on May 1 for another illness and was hospitalized on May 3. MERS-related symptoms developed on May 5 while she was hospitalized. The incubation period was compatible with either externally acquired or nosocomial infection.

**Figure 2:**
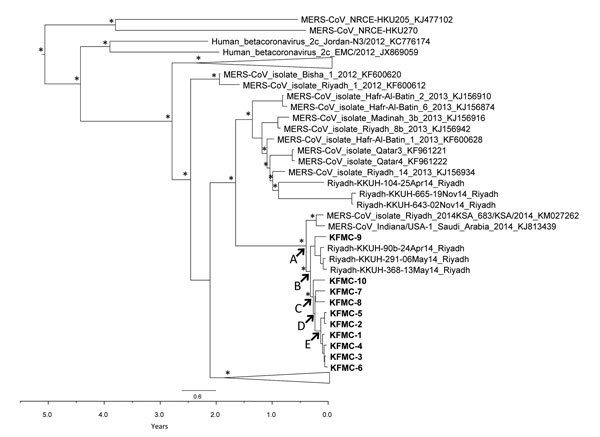
Time-resolved phylogenetic tree of Middle East respiratory syndrome coronavirus (MERS-CoV) genomes, Saudi Arabia, 2014, constructed by using BEAST version 1.8 (http://beast.bio.ed.ac.uk/). Upper scale bar indicates nucleotide substitutions per site. Lower scale bar indicates years in reference to sample KFMC-6 (collected May 18, 2014). Genomes sequenced in this study are indicated in bold. *Indicates major nodes with posterior probabilities >0.95. Estimated median dates for nodes A, B, C, D, and E (95% highest posterior density intervals) are A) Dec 31, 2013 (Nov 8, 2013–Feb 10, 2014), B) Jan 28, 2014 (Dec 16, 2013–Feb 27, 2014), C) Feb 15, 2014 (Jan 10, 2014–Mar 16, 2014), D) Feb 26,2014 (Jan 23, 2014–Mar 25, 2014), E) Apr 4, 2014 (Mar 9, 2014–Apr 25, 2014). KKUH, King Khalid University Hospital; KFMC, King Fahd Medical City.

A time-resolved phylogenetic tree (Figure 2) shows whole-genome sequences from these 10 patients within the context of other available MERS-CoV whole-genome sequences. Nodes A, B, and C have strong statistical support in this time-resolved phylogeny and in a separate maximum-likelihood phylogenetic tree of these same sequences (aBayes branch support) (*10*). Phylogenetic analysis suggests that patients at KFMC were part of a larger outbreak of MERS that was ongoing in Riyadh at that time, involving, but perhaps not limited to, other hospitals, such as PSMMC and King Khalid University Hospital (KKUH). The dated phylogeny suggests that a putative zoonotic event (node A in Figure 2) occurred on approximately December 31, 2013 (95% highest posterior density (HPD) interval November 8, 2013–February 10, 2014), although the possibility of separate zoonotic events for closely related viruses cannot be excluded.

**Figure 3:**
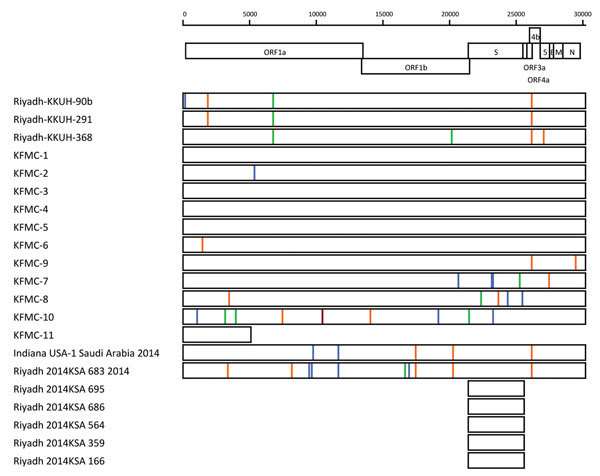
Nucleotide differences from consensus ancestral sequences of Middle East respiratory syndrome coronavirus (MERS-CoV), Saudi Arabia, 2014, estimated at nodes C and E in a time-resolved phylogenetic tree (Figure 2). The region of the genome sequenced is indicated by the length of each box. Exact genome polymorphic nucleotide positions, sampling date, and nucleotide substitutions is shown in Technical Appendix 2. Nucleotide changes are indicated by red (A), orange (T), blue (C), and green (G) vertical bars. ORF, open reading frame; KKUH, King Khalid University Hospital; KFMC, King Fahad Medical City; KSA, Kingdom of Saudi Arabia.

Virus isolate KFMC-9 clusters phylogenetically with viruses from KKUH and separately with other viruses from KFMC. This isolate has a signature mutation (C26144T KFMC-9) that is also present in KKUH-90b, KKUH-291, and KKUH-368 isolates, indicating that patient from which this virus was isolated was infected with a virus related to those in the ongoing outbreak at KKUH (Figure 3; Technical Appendix 2). The ancestral node B has strong statistical support (posterior density >0.95) and an estimated date of January 28, 2014 (95% HPD interval December 16, 2013–February 27, 2014), which is long before the date of onset of the first known case of MERS at KFMC (March 29, 2014) (Table). Node C in the dated phylogeny (Figure 2) also has strong statistical support, and an estimated date for this node was February 15 (HPD interval January 10–March 16) which is also before the date of disease onset of the first known patient in the outbreak at KFMC. Thus, it is likely that there were multiple introductions of MERS-CoV to KFMC to account for the observed virus genetic diversity in the patients studied at KFMC.

Viruses in node A in the phylogenetic tree have a nucleotide substitution rate of 6.54 × 10^−4^ nt substitutions/site/year (genome length analyzed 29,897 kb), which is comparable to a previously reported value of 6.3 × 10^–4^ (*5*). Estimated ancestral sequence at nodes C and E (identical) in the dated phylogenetic tree and nucleotide substitutions observed in virus sequences obtained in this study, together with virus sequences from patients in KKUH and PSMMC hospitals that appear to be related to this outbreak, are shown in Figure 3 and Technical Appendix 2.

We tested the hypothesis that KFMC-7, KFMC-8, and KFMC-10 viruses diverged from the ancestral virus after April 5, 2014, the date that patient EA-1 came to the ER. Observed nucleotide differences were greater than would be expected if KFMC-7, KFMC-8, and KFMC-10 diverged at KFMC after April 5, suggesting that >1 of these 3 viruses were transmitted separately to KFMC (Technical Appendix 1 Table 3). Conversely, KFMC-1–6 viruses had expected mutation rates, in accordance with observed phylogeny. Node E (including viruses KFMC-1–6) was less robust, but had an estimated date of April 4 (HPD interval March 9–April 25), which as an entry point for transmission at KFMC is more plausible with observed epidemiologic data. Viruses KFMC 1–6 had <1 nt difference between them for 29,897 nt sequenced, and the zoonotic time span between the oldest and newest virus specimens was 20 days (Technical Appendix 2). The partial genome sequence for KFMC-11 is also identical with that of KFMC-1–6 or KFMC-7. However, this partial sequence, although 5,225 nt, cannot optimally resolve transmission pathways.

### Epidemiologic Data

Ward locations and patient data are shown in Technical Appendix 1 Figure 2, and layout of key wards is shown in Technical Appendix 1 Figure 1. Before admission to KFMC, patient EA-1, the first patient to be identified during the outbreak at KFMC, had regularly visited his father, who was hospitalized at PSMMC, where a MERS outbreak was ongoing.

On the basis of known incubation periods, onset of illness, and presence at the same location (Technical Appendix 1 Figure 2), the ER was a plausible venue for MERS-CoV transmission from patient EA-1 to KFMC-0 and from patient KFMC-0 to patient KFMC-1. Patients KFMC-0 and KFMC-1 were co-workers in the ER, and patient KFMC-1 provided care for patient KFMC-0 when she was ill in the ER. Patient KFMC-1 also provided care for patient KFMC-0 without PPE in the staff quarters when she was on sick leave (April 15). There were no archived specimens from patients EA-1 and KFMC-0. Patient KFMC-1 was the first patient from this outbreak from whom we have virus genomic data.

Patient KFMC-0 was subsequently treated in MW-C where long-term patients KFMC-2 (illness onset April 25), KFMC-4 (illness onset May 1), and KFMC-6 (illness onset May 6) became ill during April 4–May 3 and were hospitalized, and viruses closely related to the virus from the KFMC-1 cluster (Technical Appendix 1 Figure 2) were isolated. Patient KFMC-3 was a chronically ill long-term patient in MW-C. A respiratory infection developed, and infection with influenza A(H1N1)pdm09 virus was detected in respiratory specimens on April 27. She was discharged on April 4 but was readmitted on May 6 because of deteriorating respiratory function and was subsequently given a diagnosis of MERS-CoV infection. Retesting of a predischarge respiratory specimen collected on April 30 showed MERS-CoV infection. Thus, patient KFMC-3 probably had MERS a few days before the testing date. However, the exact onset of illness could not be determined. Patient KFMC-3 used the intensive care unit bed previously used by patient EA-1 on April 15.

Nurse KFMC-5, who worked in MW-D, had disease onset on May 3. Virus isolated from her specimen was closely related to the cluster of viruses isolated in MW-C. Although this nurse had no duties in MW-C, MW-C and MW-D are adjacent general medical wards on the same hospital floor (Technical Appendix 1 Figure 1).

Genetic analysis suggested that viruses from patient KFMC-9, KFMC-7, KFMC-8, and KFMC-10 were introduced separately into KFMC. Patient KFMC-9 worked in the ER and patients EA-6 and EA-3, who acquired MERS outside the hospital, were admitted to the ER 4 and 11 days, respectively, before onset of disease in patient KFMC-9, which indicated that patients EA-6 and EA-3 were possible sources of infection for patient KFMC-9. Patient EA-2 was hospitalized in a 4-bed room in MW-C where nurses KFMC-7 and KFMC-10 worked. In addition, patient KFMC-8 was a long-term patient in the same ward, which provided opportunities for introduction of a genetically distinct virus (Technical Appendix 1 Figure 2).

## Discussion

We describe a hospital-associated outbreak of >45 MERS-CoV infections that occurred at KFMC, Riyadh, Saudi Arabia, during March–May 2014. There appears to be a periodicity in peaks of transmission ≈7 days apart, which is compatible with the known incubation period and case-to-case serial interval reported to be 7.6 days (*4*).

Before this molecular epidemiologic study, the assumption was that the outbreak at KFMC was self-contained and originated from patient EA-1, independent of other outbreaks reported in Riyadh. Viral genomic data obtained during this study generated alternative hypotheses and show that the outbreak of KFMC was linked to ongoing transmission within health care facilities in Riyadh at that time, including, but probably not limited to, PSMMC and KKUH. Data suggest a single zoonotic event that occurred around December 31, 2013 (95% HPD interval November 8, 2013–February 10, 2014), followed by transmission in health care facilities for ≈5 months. However, an alternative possibility of multiple, independent spillover events from closely related viruses in a zoonotic reservoir cannot be excluded. This chain of transmission was spread as far as Indiana in the United States by an HCW from Riyadh (*11*) and 2 travelers returning to the Netherlands (*12*). Viral sequence data for viruses from the 2 travelers was fragmentary and excluded from phylogenetic analysis. However, this cluster of human MERS-CoV in Riyadh was distinct from the large contemporaneous cluster of human-to-human transmission that occurred in Jeddah and represents a separate zoonotic transmission event (*6*). Only 1 of the analyzed sequences from the Riyadh cluster has an amino acid change in the receptor binding domain of the spike protein (*13*), the C23, 697T nonsynonymous mutation in KFMC-8, which leads to an R→C amino acid change.

In this outbreak, 36 cases of MERS-CoV infection were putatively acquired through nosocomial transmission. However, given ongoing human-to-human transmission in Riyadh, it cannot be ruled out that some HCWs acquired infection from outside KFMC. Molecular epidemiology indicates 1 definite cluster of transmission associated with KFMC-1–like viruses, which are genetically closely related (KFMC-1–6). There are plausible epidemiologic links for transmission from patient EA-1, the first known patient admitted to KFMC in 2014, in the ER to patient KFMC-0, then to patient KFMC-1, and to patients KFMC-2–KFMC-6. Because no virus sequence data was available patients EA-1 or KFMC-0, the role of these 2 persons in the transmission chain remains presumptive. The nearly identical virus genetic sequences for KFMC-1, -2, -3, -4, -5, and -6 and plausible epidemiologic exposures provide more definite pathways of transmission (Technical Appendix 1 Figure 2). Although virus KFMC-2 has 1 unique nucleotide substitution (T5321C), that sequence derives from a specimen collected late in the patient’s illness and might have originated in her after she transmitted infection to patients KFMC-4 and KFMC-6.

Genetic identity of virus KFMC-3 with viruses in the KFMC-1 cluster led to reassessment of the assumption that infection of patient KFMC-3 was externally acquired infection. Retesting of 2 archived (April 2014) specimens, 1 of which was positive for influenza A(H1N1)pdm09 virus, showed that patient KFMC-3 was nosocomially infected with influenza A(H1N1)pdm09 virus and MERS-CoV before her discharge on May 4, and this MERS-CoV was closely related to the KFMC-1 virus group. The source of infection for patient KFMC-3 was unclear. This patient used the intensive care unit bed used by patient EA-1 on April 15, and patient KFMC-8 occupied the isolation room vacated by patient EA-2, which raised the possibility of fomite transmission or transmission associated with HCW cases not detected by the surveillance system.

Although epidemiologic linkages would have led us to deduce that patient KFMC-9 may have acquired infection from the KFMC-1 virus cluster, viral genetic analysis conclusively demonstrates that this was a separate introduction into KFMC through a person with an externally acquired infection with a virus closely related to viruses at KKUH. Molecular epidemiology also demonstrated that virus KFMC-7, KFMC-8, and KFMC-10 were not linked to viruses in the KFMC-1 cluster, although there were plausible epidemiologic links with patients infected with viruses from the KFMC-1 cluster. These 3 infections might have resulted from 1, 2, or 3 independent virus introductions from outside KFMC.

Our data suggest that the ER and MW-C at KFMC were major foci of transmission. Although findings are not conclusive, HCWs with mild upper respiratory illness who continued to work might have contributed to transmission. Many of these issues were addressed during and after this outbreak, including, but not limited to, enhancing awareness of MERS through electronic communication, establishing in-house capacity for rapid MERS-CoV testing, active screening of KFMC staff who had influenza-like symptoms through a dedicated influenza clinic, establishing a triage area for patients in the ED, designation of wards for isolation and screening of suspected MERS cases, and strengthening infection control practices among staff by mandatory training.

Our study had limitations. Archived respiratory specimens from patients with MERS acquired outside KFMC (EA-1–EA-9) were unavailable for genomic analysis, which caused us to make assumptions in our putative chains of transmission. Some of the retrospectively retrieved epidemiologic data were obtained through interviews with HCWs and patients 1 year after the outbreak. For example, data on PPE use and extent of exposure to individual MERS-infected patients was difficult to establish with confidence. Thus, risk factors or modes of transmission (i.e., roles of large or small droplets, contact) could not be established. Dates and ward locations of patients and staff were available from the electronic medical record systems at KFMC, and we relied on proximity analysis (e.g., patients being co-housed in the same ward or nursed by the same nursing team members as other known patients with MERS) to provide epidemiologic context to the molecular epidemiologic data.

In summary, we provide molecular epidemiologic data derived from complete virus genome genetic analysis that is suggestive of a large MERS outbreak involving multiple health care facilities in Riyadh, suggesting ongoing human-to-human transmission over many months. Using molecular analysis supplemented by available epidemiologic data, we identified MERS-CoV transmission within a large health care facility and demonstrated the feasibility and value of complete viral genome sequence analysis in outbreak investigations. We showed that what was seemingly a contiguous outbreak within KFMC was caused by multiple introductions of virus from outside the hospital. The small number of mutations observed across the 29,897-nt genome analyzed during this outbreak emphasizes the need for complete genome analysis if molecular epidemiology is to be meaningful in such settings. The ongoing outbreak of MERS in South Korea (*2*), the largest cluster of transmission from a returning traveler to date, highlights the ongoing threat from MERS and the need for understanding pathways of transmission. Detailed molecular epidemiology can contribute to these efforts and thus help minimize transmission.

**Technical Appendix 1.** Additional information regarding molecular epidemiology of hospital outbreak of Middle East respiratory syndrome, Riyadh, Saudi Arabia, 2014.

**Technical Appendix 2**. Nucleotide differences from consensus ancestral sequences of Middle East respiratory syndrome coronavirus, 2014, estimated at nodes C and E of the time-resolved phylogenetic tree (Figure 2). Relevant nucleotides at each polymorphic nucleotide position, dates of sampling, number of days from nodes C and E to the time of sampling, and number of nucleotide differences from consensus sequence are indicated. Dots indicate sequence identity. UTR, untranslated region; ORF, open reading frame; KKUH, King Khalid University Hospital; KFMC, King Fahad Medical City; KSA, Kingdom of Saudi Arabia. Days of diff Node indicates days of difference to nodes C or E. *Partial sequences.
