# Supplementary material for: Molecular Epidemiology of Hospital Outbreak of Middle East Respiratory Syndrome, Riyadh, Saudi Arabia, 2014
Source: Emerg Infect Dis. 2015 Nov;21(11):1981–8. doi: 10.3201/eid2111.150944 (PMC4622263; doi:10.3201/eid2111.150944)
Supplement: Supplementary file 1 — Technical Appendix 1. Additional information regarding molecular epidemiology of hospital outbreak of Middle East respiratory syndrome, Riyadh, Saudi Arabia, 2014. [file 15-0944-Techapp-s1.pdf]

Article DOI: http://dx.doi/10.3201/eid2111.150944

Molecular Epidemiology of Hospital Outbreak of Middle East Respiratory Syndrome, Riyadh, Saudi Arabia, 2014

Technical Appendix 1

Case Definition

Persons >14 years of age suspected of being infected with Middle East respiratory syndrome virus (MERS-CoV) had to fulfill 4 criteria: 1) acute respiratory illness with clinical and/or radiologic evidence of pulmonary parenchymal disease (pneumonia or acute respiratory distress syndrome); 2) hospitalization for health care–associated pneumonia on the basis of clinical and radiologic evidence; 3) upper or lower respiratory illness ≤2 weeks after exposure to a person with a confirmed or probable case of infection with MERS-CoV; 4) acute fever (temperature ≥38°C) illness, AND body aches, headache, diarrhea, or nausea/vomiting, with or without respiratory symptoms, AND leukopenia (leukocyte count $<3.5 \times 10^9$ cells/L) and thrombocytopenia (platelet count $<150 \times 10^9$/L).

Health Care Workers

All health care workers (HCWs) who had contact with a patient suspected of having MERS or with a patient with a confirmed case of MERS were isolated and screened to determine their infection status. In March 2014, at the early stages of the outbreak, the level of alertness for MERS was not high among HCWs. There had been no previous nosocomial outbreak at KFMC. The KFMC outbreak also preceded recognition of a temporally similar outbreak in Jeddah, Saudi Arabia. Before both outbreaks, the outbreak in Al Hasa in 2013 was the only recorded major hospital outbreak of MERS and was associated with hemodialysis settings. This background might have increased the less than aggressive screening for MERS in the early stages of the outbreak at KFMC in 2014. Guidelines were more rigorously applied once evidence of an ongoing outbreak emerged in mid-April 2014.

In addition, extensive monitoring and testing of HCWs began in early May 2014. As part of this enhanced surveillance of HCWs, 5 MERS-CoV infections (4 asymptomatic and 1 mild) were detected.

Technical Appendix 1 Table 1. Characteristics of 23 health care workers and 22 other patients infected with MERS-CoV, King Fahad Medical City, Saudi Arabia, 2014*

	No. (%)	
Characteristic	Health care worker	Other
Sex		
M	5 (22)	15 (68.2)
F	18 (78)	7 (31.8)
Age, y		
<20	0	1 (4.5)
20–29	3 (13.0)	3 (13.6)
30–39	13 (56.5)	2 (9.0)
40–49	4 (17.4)	1 (4.5)
≥50	3 (13.0)	15 (68.2)
Job category		
Nurse	18 (78)	NA
Respiratory therapist	3 (13)	NA
Pharmacist	1 (4)	NA
Radiology technician	1 (4)	NA
Job location		
Critical care	4 (18)	NA
Emergency department	8 (35)	NA
Medical ward	9 (39)	NA
Pharmacy	1 (4)	NA
Radiology	1 (4)	NA

MERS-CoV, Middle East respiratory syndrome coronavirus; NA, not applicable.

Technical Appendix 1 Table 2. Demographic, laboratory, and sequence data for selected patients with suspected MERS-CoV infections, King Fahad Medical City, Saudi Arabia, 2014*

Patient	Age, y/sex	Date of illness onset	Patient group	Date of specimen used for viral genomic analysis	C_t value of specimen used for viral genomic analysis	No. (%) nucleotides of MERS-CoV genome sequenced†	GenBank accession no.
Externally acquired infections							
EA1	32/M	Mar 29	Externally acquired	NA	–	–	–
EA2	65/F	Apr 6	Externally acquired	NA	–	–	–
EA3	46/F	Apr 13	Externally acquired	NA	–	–	–
EA4	70/M	Apr 18	Externally acquired	NA	–	–	–
EA5	64/M	Apr 18	Externally acquired	NA	–	–	–
EA6	22/F	Apr 20	Externally acquired	NA	–	–	–
EA7	28/F	May 1	Externally acquired	NA	–	–	–
EA8	21/F	May 5	Externally acquired	NA	–	–	–
EA9	50/F	May 5	Externally/hospital acquired‡	NA	–	–	–
Nosocomial infections							
KFMC-0	34/F	Apr 9	ED nurse	NA	–	–	–
KFMC-1	45/F	Apr 20	ED nurse	Apr 28	24.96	29,897 (99.3)	KT121580
KFMC-2	60/F	Apr 25	Patient	May 11	30.16	29,897 (99.3)	KT121577
KFMC-3	62/F	Apr 27	Patient	May 9	29.64	29,897 (99.3)	KT121573
KFMC-4	63/F	May 1	Patient	May 12	30.80	29,897 (99.3)	KT121575
KFMC-5	56/F	May 3	Nurse MW-D	May 12	28.34	29,897 (99.3)	KT121572
KFMC-6	74/F	May 6	Patient	May 18	28.99	29,897 (99.3)	KT121576
KFMC-7	36/F	Apr 26	Nurse MW-C	May 3	30.94	29,897 (99.3)	KT121581
KFMC-8	53/F	Apr 30	Patient	Apr 30	31.73	29,897 (99.3)	KT121579
KFMC-9	29/M	May 1	ED nurse	May 12	29.84	29,897 (99.3)	KT121574
KFMC-10	46/F	Apr 23	Nurse MW-C	May 15	32.13	29,897 (99.3)	KT121578
KFMC-11	41/F	Apr 24	Nurse MW-C	Apr 28	35.78	5,225 (17.5)	KT202801

*MERS-CoV, Middle East respiratory syndrome coronavirus; C_t, cycle threshold; NA, not available; –, not applicable; ED, emergency department; MW, medical ward.
†Full genome of MERS-CoV is 30,106 nt.
‡This patient came to the ED on May 1, 2014, because of another illness and was hospitalized on May 3. MERS-related symptoms developed on May 5 during hospitalization. The incubation period was compatible with an externally acquired infection or a nosocomial infection.

Technical Appendix 1 Table 3. Compatibility of observed MERS-CoV genome variation from ancestral genome sequence and virus genome mutation rates from date of admission (April 5) of the first known MERS patient at KFMC, Saudi Arabia, 2014*

Virus isolate	Time, d	No. substitutions	No expected substitutions	p-value†
KFMC-10	26	9	1.394	0.0000158
KFMC-7	23	5	1.233	0.0086
KFMC-8	25	5	1.34	0.012

*KFMC, King Fahad Medical City. MERS-CoV, Middle East respiratory syndrome coronavirus. The estimated virus mutation rate was 6.54×10^{4} nucleotides/site/year across a sequence length of 29,897 nt.
†Refers to the cumulative probability that the mean expected number of substitutions is greater than or equal to the observed number of substitutions. p-values for accepting or rejecting the null hypothesis were significant at the 0.05 level after using the Bonferroni correction to adjust the p-value for each hypothesis to p<0.0167.

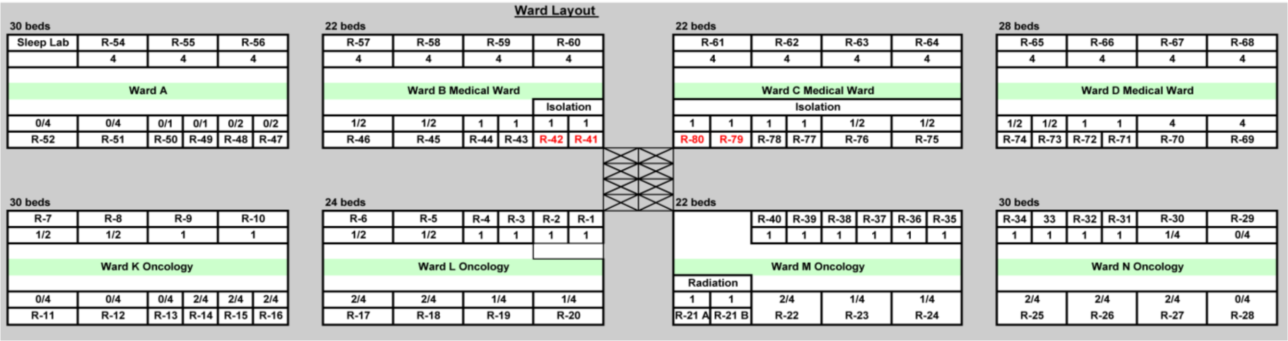

Technical Appendix 1 Figure 1. Layout of most relevant hospital wards at King Fahad Medical City, Riyadh, Saudi Arabia, 2014.

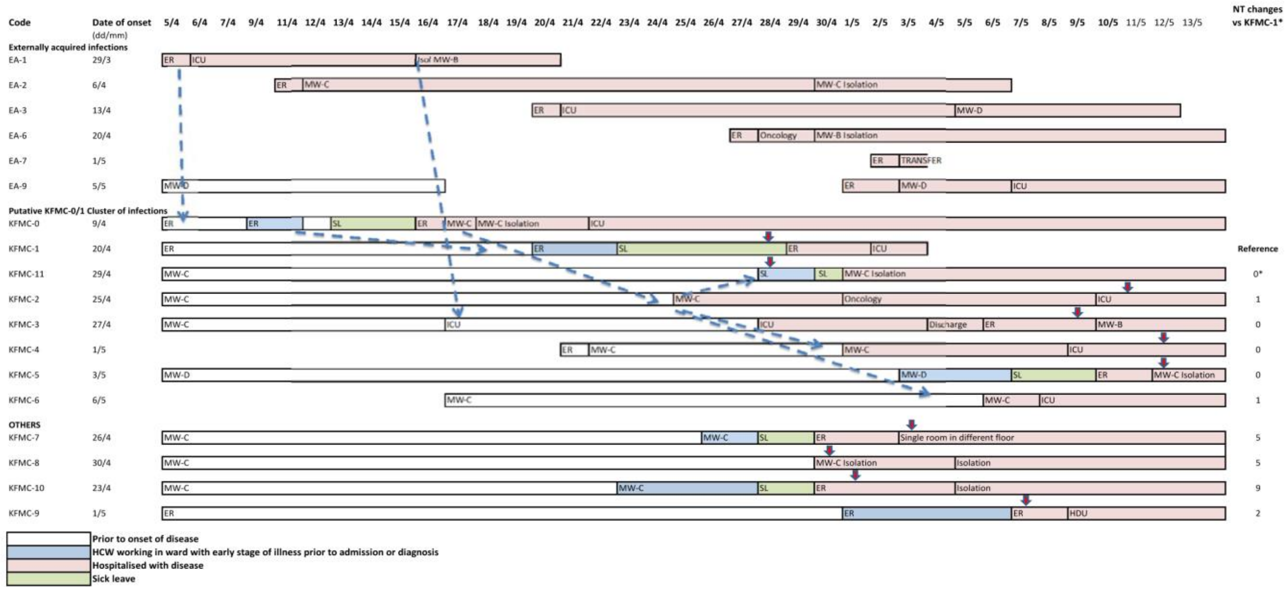

Technical Appendix 1 Figure 2. Locations of patients during an outbreak of Middle East respiratory syndrome, King Fahad Medical City (KFMC), Riyadh, Saudi Arabia, 2014. Locations of persons before onset of MERS are indicated by white rectangles. Locations of persons at or after time of onset of illness are indicated by pink rectangles. For infected health care workers (HCWs), the period between onset of illness and hospital emergency room (ER) attendance or hospital admission during which time they might have been still working in their relevant wards is indicated by blue rectangles.

Periods of sick leave (SL) are indicated by green rectangles. Vertical arrowheads indicate time of collection of samples used in genomic analysis. For ease of reference, contacts and transmission pathways involving the KFMC-1 cluster discussed in the text are indicated by blue dashed arrows. These arrows are not meant to imply definitive transmission pathways but are for ease of reference only. *Indicates number of nucleotide changes with reference to KFMC-1 across the entire genome of 29,897 nt, except for KFMC-11, in which only 5,225 nt are available. ICU, intensive care unit; MW, medical ward; HDU, high dependency unit.
